# Shouting strengthens voluntary force during sustained maximal effort through enhancement of motor system state via motor commands

**DOI:** 10.1038/s41598-022-20643-4

**Published:** 2022-09-28

**Authors:** Yudai Takarada, Daichi Nozaki

**Affiliations:** 1 Faculty of Sports Sciences, Waseda University, 2-579-15 Mikajima, Tokorozawa, Saitama 359-1192 Japan; 2 Graduate School of Education, The University of Tokyo, Tokyo, 113-0033 Japan

**Keywords:** Neuroscience, Physiology, Psychology

## Abstract

Previous research indicates that shouting during momentary maximal exertion effort potentiates the maximal voluntary force through the potentiation of motor cortical excitability. However, the muscular force-enhancing effects of shouting on sustained maximal force production remain unclear. We investigated the effect of shouting on the motor system state by examining motor evoked potentials in response to transcranial magnetic stimulation applied over the hand area of the contralateral primary motor cortex (M1) during sustained maximal voluntary contraction, and by assessing handgrip maximal voluntary force. We observed that shouting significantly increased handgrip maximal voluntary force and reduced the silent period. Our results indicate that shouting increased handgrip voluntary force during sustained maximal exertion effort through the reduced silent period. This is the first objective evidence that the muscular force of shouting during maximal force exertion is associated with the potentiation of motor system activity produced by the additional drive of shouting operating on the motor system (i.e., shouting-induced excitatory input to M1).

## Introduction

It has been established that force levels of maximal voluntary contraction (MVC), in which a person believes their effort to be maximal, fluctuate constantly. Ikai and Steinhaus^1^ proposed that the MVC is limited by psychological inhibiting mechanisms. The maximal voluntary force has been found to be increased by various manipulations such as the sound of a gunshot^1^, hypnotic suggestion^1^, shouting^1,2^, verbal encouragement^3^, and motivational goal-priming^4^. However, the mechanisms underlying this enhancement effect on force production remain unclear.

Recently, we found that a self-generated shout increased the handgrip maximal voluntary force during brief non-fatiguing maximal effort through reduced motor cortical inhibition, associated with the potentiation of pupil-linked neuromodulatory system activity^5^. Additionally, motor cortical inhibition in fatigued muscle has been reported during sustained or repeated maximal effort, and the strength of this inhibition is substantially greater than that in unfatigued muscle during brief non-fatiguing maximal effort^6^. If shouting has a motor cortical inhibition-reducing effect, as found during a brief MVC in brief non-fatiguing maximal effort^5^, it would be expected to reduce motor cortical inhibition during sustained MVC in fatiguing maximal effort. This hypothesis is supported by previous evidence that shouting increases the maximal voluntary force of fatigued muscle during repeated maximal effort once a minute for 30 min^1^.

Of note, in the shouting protocols used to increase MVC in several previous studies^1,2,5^, a self-generated shout occurred immediately prior to or almost simultaneously with muscular force exertion. Thus, shouting-induced excitatory input to the motor system did not occur after muscular force exertion in those studies, making it impossible to examine the effect of shouting-induced motor commands on the motor system state as distinct from the effects of force exertion-induced motor commands. We further hypothesize that shouting following force exertion in the above sustained maximal effort would be expected to enhance the maximal voluntary force through potentiation of the motor system because we consider that producing a shout in itself is a requisite for the muscular force-enhancing effect of shouting.

In the present paper, we investigate the effect of shouting on the motor system state by examining motor evoked potentials (MEPs) in response to transcranial magnetic stimulation (TMS) applied over the hand area of the contralateral primary motor cortex (M1) during sustained MVC with intermittent shouting following force exertion. We also investigate the pupil-linked neuromodulatory system state by examining the pupil size^7,8^ and by assessing the handgrip maximal voluntary force. Our results indicate that shouting can increase the force level of MVC through the reduction of motor cortical inhibition during sustained MVC. This is the first objective evidence that the muscular force-enhancing effect of shouting in maximal force exertion is associated with the enhancement of motor system activity, produced by the additional drive of shouting operating on the motor system (M1). Therefore, such new insight into the mechanism underlying the shout-induced enhancement effect on force production will have substantial benefits. In other words, we will be able to make use of the muscular force-enhancing effects of shouting on the maximal force production by a conscious self-generated shout based on the mechanism, when we must exert a muscular force with the maximal effort or when we must make one last effort as we accept our limitations. Additionally, athletes and/or coaches will be able to consciously make use of the muscular force-enhancing effects of shouting on maximal force production for improvement of performance.

## Methods

### Power analysis

We conducted an a priori power analysis to determine the required sample size for the experiment. We designed the experiment to have 80% power for detecting the effect sizes that we previously found for the effect of motivational goal-priming on the motor system and action (0.46–0.64, Cohen’s *d*)^4,5,7^ and/or pupil diameter (0.50–0.61, Cohen’s *d*)^7,8^, using a significance level of 5%. We used G*Power 3.1® (Institut für Experimentelle Psychologie, Düsseldorf, Germany) to compute the required total sample size of the current study by conducting a repeated-measures analysis of variance (ANOVA) with within—between interaction (within-participants factor [shouting or no shouting])^2^ and between-participants^2^ factors (experimental group [shouting in the odd-numbered TMS trials or shouting in the even-numbered TMS trials]) for 16 participants, using 80% power (1 − β error probability). Thus, the sample size of each experimental group was 8 participants.

### Participants and procedures

Sixteen healthy Japanese right-handed individuals, as evaluated using the Edinburgh Handedness Inventory^9^, participated in the study. The participants were 16 men, with a mean age ± standard deviation of 20.1 ± 1.6 years. All participants provided both written and verbal informed consent. The study was conducted in accordance with the Declaration of Helsinki. All participants were university students who reported no strength training history, indicating that they had not received training regarding the exertion of the maximal force generated briefly by a muscle or group of muscles at a specified speed. Participants were randomly assigned to one of two groups (n = 8 for each group), one of which was for shouting in the odd-numbered (first) TMS trials and the other was for the even-numbered (later) TMS trials in a 2-min sustained MVC with five periods of intermittent shouting. Each group underwent two (shouting only and MVC) tasks of tests consecutively with at least a 3-min interval between tasks. The experimental procedures complied with relevant laws and institutional guidelines and were approved by the Human Research Ethics Committee of the Faculty of Sport Sciences of Waseda University (Approval Number: 2020-411).

**Figure 1:**
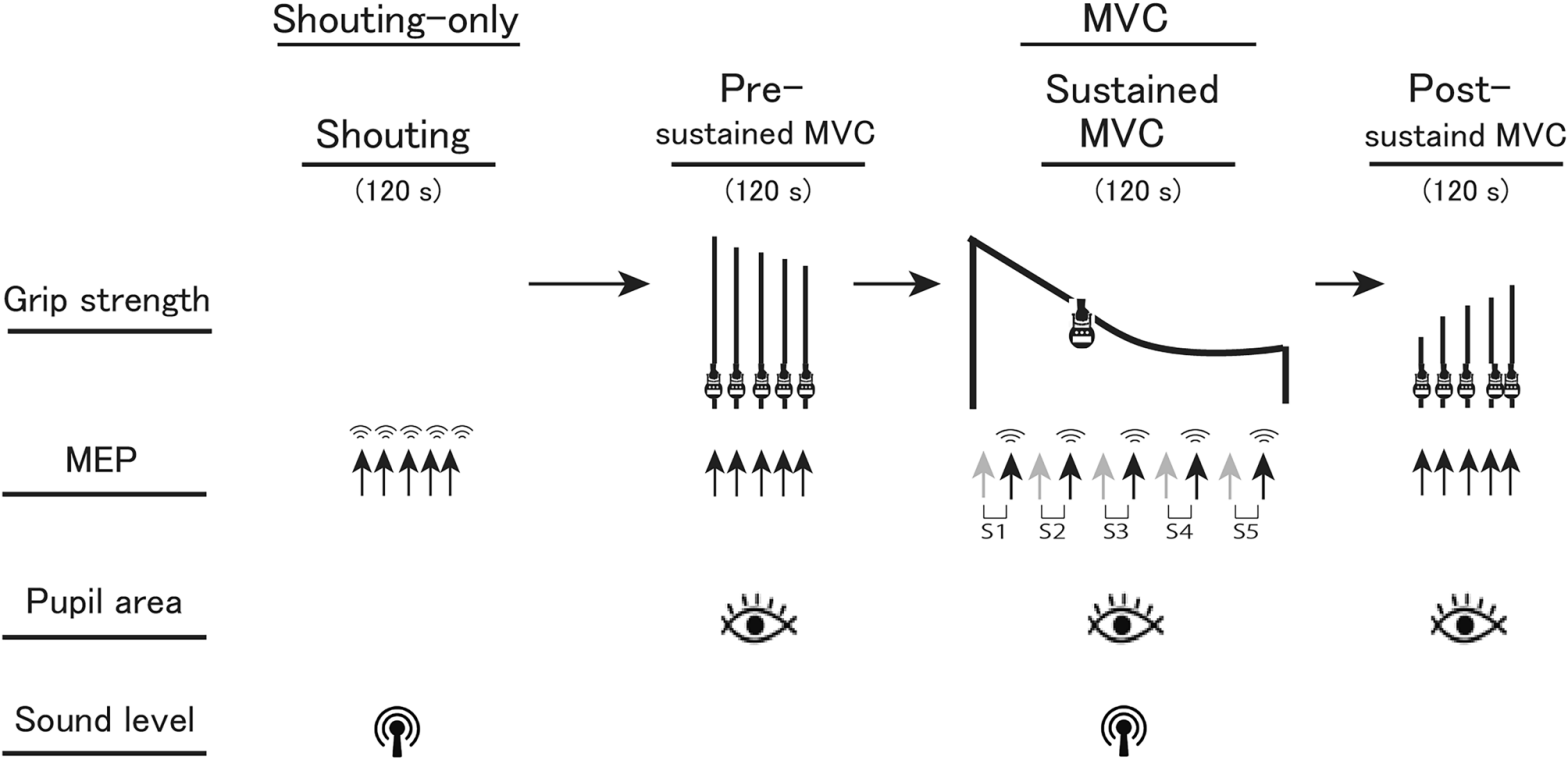
Experimental procedure. Each experiment consisted of two tasks (only shouting and MVC). The MVC task consisted of three conditions (pre-sustained MVC, sustained MVC, and post-sustained MVC), each with a duration of approximately 120 s, which were performed in that order with a break of at least 3 min between conditions. The total experimental time was approximately 20 min. The timing of transcranial magnetic stimulation (TMS) is indicated by the arrow. Ten TMS during the sustained MVC condition were classified into the five sets (S1 ~ S5) according to the timeline. The order of shouting in the sustained MVC condition was counterbalanced so that eight participants shouted in the odd-numbered TMS trials (Experimental group 1) and the other participants shouted in the even-numbered TMS trials (Experimental group 2).

Experiments were designed to examine the effect of a self-generated shout on the handgrip maximal voluntary force, pupillary size, and MEPs in the flexor carpi ulnaris (FCU) muscle in response to TMS (see “TMS” for details). Each experiment consisted of two tasks (shouting only and MVC), which were spaced at least 3 min apart on the same day. The MVC task was performed under three conditions (in the order of pre-sustained MVC, sustained MVC, and post-sustained MVC conditions), each lasting approximately 120 s (Fig. 1). In the shouting-only task, participants were asked to shout five times with a 30-s inter-shouting interval, as loudly as possible for 1–2 s when a cue was given by an experimenter (“One, two, three, start”) in a quiet voice. In the pre-sustained MVC condition of the MVC task as a baseline of the sustained MVC condition, participants performed five brief MVCs (1–2 s duration) when an experimenter gave a cue (“One, two, three, squeeze”) with a 30-s inter-squeeze interval. After a rest of approximately 3 min, participants performed a 2-min sustained MVC with five periods of intermittent shouting (sustained MVC condition) according to experimental instructions displayed on a screen in front of them (see “Pupil diameter measurement” for details). A previous study reported that 2-min sustained MVC induces a progressive, exercise-induced decline in the level of motoneuronal drive during a muscular contraction (central fatigue)^6^. Participants were not given advance notice of when the sustained MVC condition would terminate. However, they were informed in advance that they would be asked to shout five times with a 24-s inter-shouting interval (see “TMS” for details) during the sustained MVC condition (Fig. 1) by an experimenter, as in the shouting-only task. Immediately following the sustained MVC, a series of brief contractions was performed with maximal effort. This was the same process as that of the pre-sustained MVC condition (post-MVC condition). The total experimental period was approximately 20 min. Visual feedback and verbal encouragement were given throughout all handgrip contractions. Under all conditions, participants were asked to keep their heads still and to keep their hands on their lap in a sitting posture while maintaining as much stability in the core as possible, and to keep viewing the screen in front of them.

### Pupil diameter measurement

The pupil diameter was measured using a TalkEye Lite system (Takei Scientific Instruments Co., Ltd., Tokyo, Japan). An image around the pupil was obtained using a camera employing near-infrared light-emitting diodes and a video graphics array (640 × 480) (built-in digital signal processor) camera module (NCM03-V, Nippon Chemi-Con Corporation, Tokyo, Japan). Banalization processing was performed on each image, and the pupil diameter was then measured according to the methods described by Wang et al.^10^. Changes in pupil size were estimated by the area of the pupil^7,8^ while participants viewed the screen in front of them under all conditions. We calculated the average pupil area (dots) for 500 ms before each TMS (see “TMS”) during squeezing of a handgrip device (see “Handgrip force measurement”) under the pre-sustained MVC, post-sustained MVC, and sustained MVC conditions of the MVC task.

The following steps were taken to exclude the effect of experimenter expectations for participant responses and measurements as much as possible, and to objectively estimate the effect of shouting under the sustained MVC condition. (1) The experimental procedure of the sustained MVC condition was conducted automatically using a 60-Hz cathode ray tube screen to display the text, and the experimental procedure was created using software designed for psychological experiments (Inquisit 3 Desktop Edition, Millisecond Software, Seattle, WA, USA). (2) All participants were instructed to follow starting and stopping signals on the screen. (3) Pupil diameter measurements were automatically performed using a specially designed device with an eye-capturing camera to obtain an image around the pupil. Consequently, the paradigm used in the present study was less susceptible to experimenter bias compared with outcome measurements that have typically been used for examining the maximal voluntary force in previous studies^11^.

All word stimuli were displayed in black (20.5 cd/m^2^: mean value of five measurements of luminance with an LS160 luminance meter; Konica Minolta, Inc., Tokyo, Japan) on a white screen (123.2 cd/m^2^) in the experimental procedure. Immediately before the word presentation, the color of the screen was momentarily white without any black words. The pupil diameter may have transiently decreased because of the increase in luminance caused by the white screen with a maximum luminance of 128.5 cd/m^2^. Thus, we were unable to completely eliminate the possibility that this transient change in luminance affected the pupil diameter. However, any effect on the results would likely be minimal because this phenomenon was present for all participants and conditions.

### Handgrip force measurement

**Figure 2:**
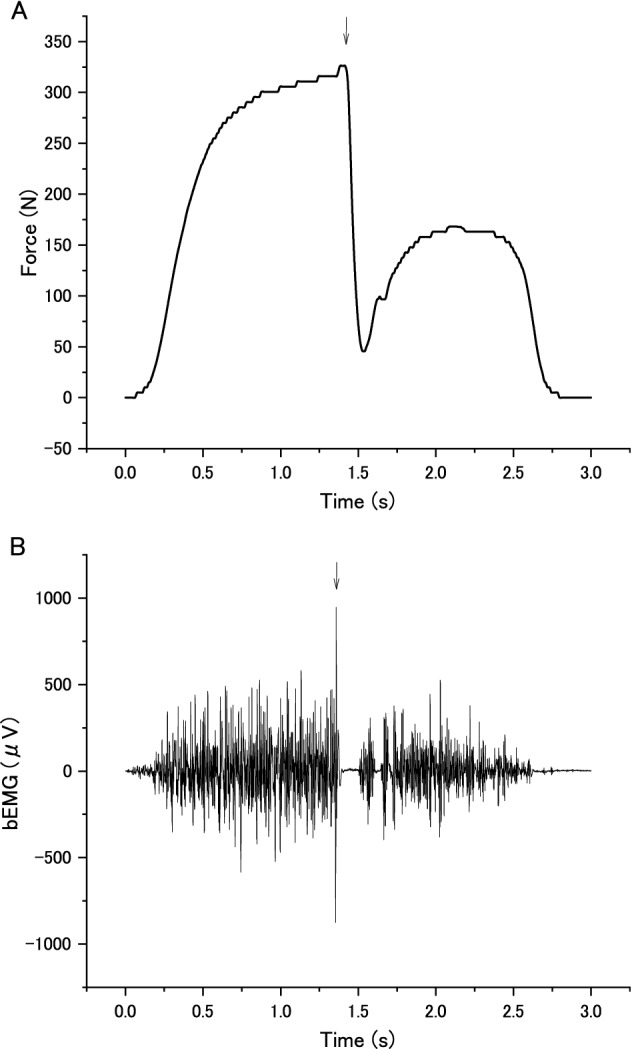
Typical recordings of handgrip force, and background electromyography (bEMG) of the flexor carpi ulnaris during the maximal voluntary contraction (MVC) of handgrip in pre-sustained MVC condition in a single participant. The timing of transcranial magnetic stimulation is indicated by the arrow. The handgrip force declined when transcranial magnetic stimulation was delivered during the contraction, the timing of which was different in each contraction. (**A**) Handgrip force. (**B**) bEMG during handgrip contraction.

The force was measured using a handgrip device (KFG-5-120-C1-16, Kyowa Electronic Instruments, Tokyo, Japan). Under the pre- and post-sustained MVC conditions, participants performed five brief MVCs (1–2 s duration) on a cue given by an experimenter (“One, two, three, squeeze”) with a 30-s inter-squeeze interval. Under the sustained MVC condition, the experimental instructions displayed on the screen asked participants to squeeze the handgrip device with the right (dominant) hand with maximum effort when the word “squeeze” appeared on the display, and to stop squeezing when the word disappeared. The handgrip device was fixed to the right thigh with an elastic band so that the device did not move when it was squeezed by the participant. The maximal values of the exerted force were averaged from the 500-ms steady state of the force curve before each TMS (Fig. 2A,B) across the five trials for the pre- and post-MVC conditions, and in each trial for the sustained MVC condition (see “TMS” for details). These averages were taken as the handgrip maximal voluntary force.

### TMS

In the shouting-only task, the pre-sustained MVC, sustained MVC, and post-MVC conditions of the MVC task single-pulse TMS were administered via a stimulator (M2002, Magstim, Whitland, UK) using a double-figure-eight-shaped coil (4150-00 Double 70 mm Alpha Coil, Magstim) with a maximum magnetic field strength of 1.55 T. Each participant sat upright with their elbows bent in front of them, and their hands resting on their thighs. The TMS coil was then positioned over the finger area of the left M1, which was determined as the area with the lowest resting motor threshold (rMT). This was defined as the area for which MEPs with peak-to-peak amplitudes greater than 50 µV were induced in the FCU muscle^5,7,12,13^ in at least five of 10 trials when participants were fully relaxed with their eyes closed^14^. The coil position was stabilized throughout the experiment using a coil stand made from multiple products (Manfrotto Distribution KK, Tokyo, Japan). The optimal scalp position of M1 was marked directly onto the scalp with a black marker pen. The positioned coil was monitored continuously to maintain consistent positioning throughout the experiment. The rMTs ranged from 40 to 80% of the maximum stimulator output, and the stimulus intensity for each participant was set at almost the same intensity as their rMT while shouting in the shouting-only task. The stimulus intensity was set from 70 to 90% of the maximum stimulator output during handgrip force exertion. Stimulation was manually delivered over the target site during each shouting period (1–2 s duration) and each brief MVC (1–2 s duration), with a 30-s inter-squeeze interval (Fig. 2A,B). The timing of TMS was different for each episode of shouting under the shouting condition of the shouting-only task, and each brief MVC under the pre- and post-MVC conditions of the MVC task. Thus, MEPs were recorded five times for each condition (shouting or pre- or post-sustained MVC). The stimulation was automatically delivered 10 times at 12-s intervals for the 2-min sustained MVC condition, during which shouting (sustained MVC condition with shouting) and no shouting (sustained MVC condition without shouting) were alternated every 12 s. Thus, MEPs were recorded 10 times. Ten MEPs were classified into five sets (S1–S5) according to the timeline, each including two MEPs with or without shouting (Fig. 1). The order of the shouting under the sustained MVC condition was counterbalanced so that eight participants shouted in the odd-numbered TMS trials (Experimental group 1) and the other participants shouted in the even-numbered TMS trials (Experimental group 2). The TMS intensity was set from 70 to 90% of the maximum stimulator output for each participant. Surface electromyography (EMG) was obtained from the right FCU muscles via bipolar silver surface electrodes (10 mm in diameter) using the tendon-belly method^5,7,13^. The skin overlying the identified muscles was cleaned with alcohol pads prior to electrode placement. Signals (analysis time of 30 ms) were amplified using a bandpass filter (15 Hz–10 kHz) and digitized (MEG-6108; Nihon Kohden Co., Tokyo, Japan) at a sampling rate of 4 kHz.

### Background EMG and MEP measurement and analysis

**Figure 3:**
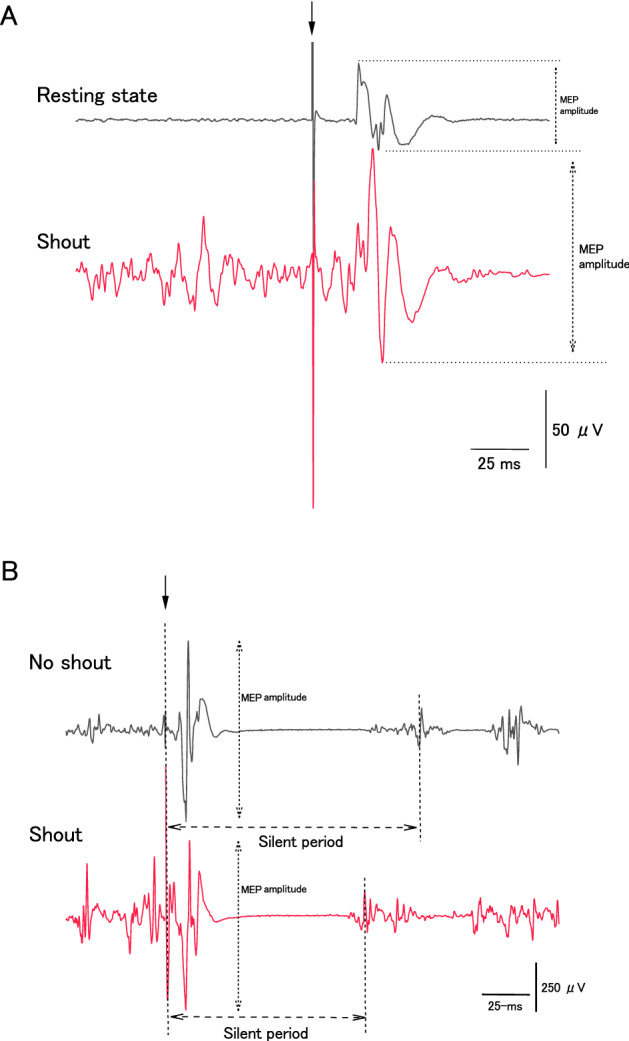
Typical motor evoked potential (MEP) waveforms of the flexor carpi ulnaris (FCU) during the maximal voluntary contraction (MVC) of handgrip in a single participant. The timing of transcranial magnetic stimulation is indicated by the arrow. MEPs measured from the relaxed FCU muscle during the resting state and during shouting for the shouting-only condition in a single participant (**A**) and during handgrip contraction with or without shouting for the sustained MVC condition in a single participant (**B**). The bidirectional arrows indicate amplitudes of MEPs and duration of the silent period.

We measured the peak-to-peak amplitude of each MEP (Fig. 3A,B). We calculated the averaged waveform of the MEP (an average of five recordings) in the shouting-only task, the pre-sustained MVC, sustained MVC, and post-MVC conditions of the MVC task (see “TMS” for details). To measure the EMG background (bEMG), a rectified EMG signal having a period of approximately 100 ms before TMS was integrated, with the force kept at the maximum force level (Fig. 2A,B). These analyses were performed with analysis software (LabChart 7.3.8; ADInstruments, Tokyo, Japan).

The duration of the silent period was taken as the time interval from the stimulus artifact to the return of continuous EMG^15,16^ (Fig. 3B). When it was difficult to determine the end of the silent period (because voluntary EMG activity recovers gradually rather than abruptly), the end of the silent period was determined as the moment that the corresponding rectified EMG activity reached a value within two standard deviations of the rectified EMG signal in the period approximately 100 ms before TMS^17,18^.

### Sound level

Sound level in the shouting condition and the sustained MVC condition was measured using by a Digital Sound Level Meter (GM1356, Benetech, Guangdong, China), which was placed diagonally in front of the participant and to their left, at face level. The readings are in Decibel, A-weighted (dBA) units representing the sound level measured with the A-weighting network on the sound level meter. This instrument incorporates a type-1 microphone and records sound ranging from 30 to 130 dBA with a sensitivity index of ± 0.1 dBA (sampling rate 20 Hz). Fast impulse mode was used to record the readings.

### Statistical analysis

MEP data of the resting state for the rMT measurement and only shouting conditions in the only shouting task were analyzed in repeated-measures two-way ANOVAs with a between-participant factor of Group (first or later) and a within-participant factor of Condition (resting state for the rMT measurement or only shouting). Additionally, sound level data of only shouting in the only shouting task and the sustained MVC conditions in the MVC task were analyzed in repeated-measures two-way ANOVAs with a between-participant factor of Group (first or later) and a within-participant factor of Condition (only shouting or a 2-min sustained MVC with five periods of intermittent shouting). The maximal voluntary force, the duration of the silent period, MEP, bEMG, and pupil area under the sustained MVC condition were analyzed in repeated-measures three-way ANOVAs of the experimental group (between-participants factor: shouting in the odd-numbered TMS trials [later: group 1] or shouting in the even-numbered TMS trials [first: group 2] [2]) × order of each set (within-participants factor: [shouting or no shouting]) (2) × set (within-participants factor: [1–5 sets] [5]). Greenhouse–Geisser corrections were applied when appropriate to adjust for non-sphericity, changing the degrees of freedom using a correction coefficient. A significance threshold of *p* < 0.05 was chosen for all tests. When the results of the main effect and interaction of the three-way ANOVA are presented, partial η^2^ (η_p_^2^) is also shown as an effect size index. The values of η_p_^2^ were interpreted as 0.07–0.23, 0.24–0.59, and ≥ 0.6 for small, medium and large effects, respectively^19^.

## Results

We first ascertained that shouting enhanced the excitability of motor system activity in the only shouting task: shouting (experimental group first: 208.92 ± 61.03 µV; later: 190.36 ± 51.39 µV) significantly increased MEP amplitudes, relative to those of rMT (experimental group first: 68.07 ± 9.66 µV; later: 87.44 ± 19.53 µV) {Condition (resting state vs. only shouting): *F*[1,14] = 11.45; *p* = 0.004; effect size: η^2^_p_ = 0.45); Interaction between Condition and Group: *F*[1,14)] = 0.27; *p* = 0.60; effect size: η^2^_p_ = 0.019)} (Fig. 3A). There were no significant differences in the sound level between the shouting-only (experimental group first: 106.74 ± 1.97 dBA; later 104.66 ± 1.00 dBA) and sustained MVC conditions (experimental group first: 105.86 ± 1.85 dBA; later 103.38 ± 1.13 dBA) (Condition (only shouting vs. a 2-min sustained MVC with intermittent shouting): *F*[1,14] = 3.32; *p* = 0.09; effect size: η^2^_p _= 0.19); Interaction between Condition and Group *F*[1,14] = 0.11; *p* = 0.73; effect size: η^2^_p_ = 0.008).

Several effects of shouting on behavior and the motor system state were observed (Table 1); these were generally consistent with previously published findings^5^. Shouting significantly increased the handgrip maximal voluntary force and was followed by a reduced duration of the silent period.

**Table 1:** Maximal voluntary force, silent period, MEP amplitude, bEMG and pupil area for the four conditions.

	Maximal voluntary force (N)	Silent period (ms)	MEP amplitude (mV)	bEMG (μV s)	Pupil area (dot)
**Experimental group (later)**					
Pre-MVC	282.2 ± 18.5	157.9 ± 10.5	1.12 ± 0.10	13.5 ± 1.41	2807.0 ± 159.2
Sustained MVCs					
Set1 no-shout	261.9 ± 23.3	155.1 ± 16.2	1.80 ± 0.19	10.5 ± 0.85	2696.9 ± 204.3
Shout	240.7 ± 17.0	152.5 ± 19.9	1.58 ± 0.17	10.9 ± 1.18	2526.2 ± 141.0
Set 2 no-shout	155.3 ± 16.1	209.2 ± 15.6	1.92 ± 0.20	9.33 ± 2.09	2502.3 ± 199.1
Shout	162.6 ± 17.0	163.3 ± 25.9	1.68 ± 0.30	9.35 ± 2.46	2415.8 ± 203.2
Set 3 no-shout	128.1 ± 14.0	191.7 ± 26.0	1.70 ± 0.27	7.97 ± 2.58	2542.3 ± 196.9
Shout	139.4 ± 12.2	181.0 ± 32.7	1.51 ± 0.23	9.34 ± 2.60	2227.2 ± 145.6
Set 4 no-shout	106.9 ± 8.1	265.9 ± 71.6	1.78 ± 0.22	9.51 ± 2.93	2561.4 ± 166.1
Shout	123.0 ± 12.3	165.1 ± 32.0	1.59 ± 0.30	11.4 ± 1.87	2398.2 ± 159.8
Set 5 no-shout	93.9 ± 7.4	180.4 ± 16.5	1.76 ± 0.27	6.20 ± 1.78	2655.0 ± 185.0
Shout	106.0 ± 10.5	165.3 ± 21.3	1.62 ± 0.22	7.83 ± 1.64	2148.2 ± 199.3
Post-MVC	200.7 ± 19.6	151.7 ± 12.0	1.13 ± 0.09	12.0 ± 1.46	2748.6 ± 138.6
**Experimental group (first)**					
Pre-MVC	298.7 ± 17.5	178.9 ± 18.5	0.72 ± 0.10	9.73 ± 2.07	2401.0 ± 171.4
Sustained MVCs					
Set1 no-shout	217.5 ± 23.9	257.1 ± 65.5	1.05 ± 0.13	8.21 ± 1.00	2315.0 ± 177.4
Shout	301.0 ± 30.6	165.0 ± 20.6	0.92 ± 0.24	10.54 ± 1.76	2521.8 ± 173.1
Set 2 no-shout	158.3 ± 14.3	222.7 ± 40.0	1.16 ± 0.14	5.39 ± 1.47	2349.9 ± 171.4
Shout	207.4 ± 27.5	170.8 ± 20.7	1.13 ± 0.12	7.83 ± 1.48	2327.5 ± 122.6
Set 3 no-shout	120.3 ± 11.9	180.2 ± 21.9	0.97 ± 0.20	3.78 ± 0.88	2241.0 ± 166.1
Shout	185.3 ± 25.8	163.7 ± 17.8	1.12 ± 0.16	7.71 ± 2.35	2487.9 ± 152.1
Set 4 no-shout	102.7 ± 7.05	176.2 ± 26.1	0.98 ± 0.097	2.61 ± 0.50	2178.8 ± 151.3
Shout	159.5 ± 19.8	188.3 ± 16.6	0.93 ± 0.082	6.96 ± 1.67	2375.8 ± 207.0
Set 5 no-shout	87.6 ± 5.7	229.0 ± 23.8	1.02 ± 0.080	3.50 ± 0.57	2286.3 ± 236.6
Shout	126.0 ± 15.5	163.7 ± 20.3	1.15 ± 0.16	4.77 ± 1.44	2360.2 ± 218.2
Post-MVC	199.3 ± 15.5	162.4 ± 18.1	0.91 ± 0.10	7.65 ± 0.80	2333.7 ± 198.4

*MEP* motor evoked potential, *MVC* maximal volutanry contration, *bEMG* background electromyography.

### Handgrip force

**Figure 4:**
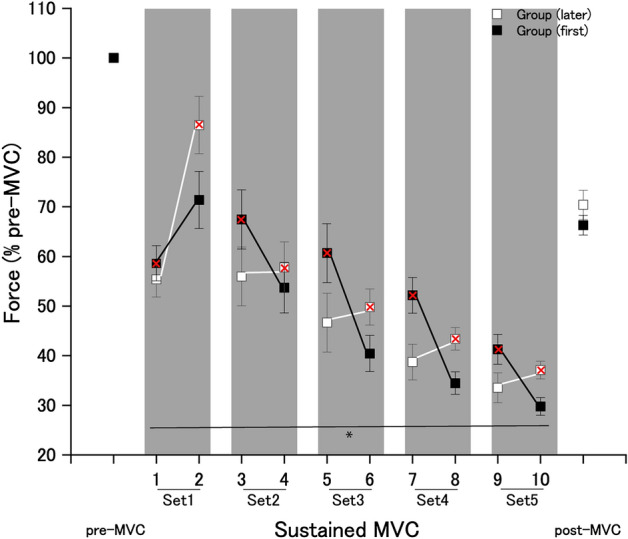
Effects of shouting on the maximal voluntary force of handgrip. The maximal voluntary force for each condition (pre-sustained MVCs, sustained MVC, and post-sustained MVCs conditions) during the maximal voluntary contraction (MVC) of handgrip in each experimental group. Shouting significantly increased the maximal voluntary force of handgrip during a 2-min sustained MVC. Red cross indicates handgrip contraction with shouting. *Statistically significant difference between the handgrip contractions performed with and without shouting in shaded area. Data are expressed as the mean ± standard error of the mean.

Significant changes in the maximal voluntary force during the sustained MVC were observed when combined with shouting. Three-way ANOVA revealed that there was no significant second-order interaction (*F*[4,56] = 0.31; *p* = 0.86; effect size: η^2^_p _= 0.02) (Table 2). There were significant first-order interactions between the order of sets (shouting or no shouting) and the experimental group (*F*[1,14] = 13.23; *p* = 0.003; effect size: η^2^_p_ = 0.48) and between the set (sets 1–5) and the order of each set (shouting or no shouting) (*F*[4,56] = 14.00; *p* < 0.001; effect size: η^2^_p_ = 0.50). There were significant simple main effects of shouting on the maximal voluntary force in each experimental group (order of shouting [first]: *p* = 0.016; effect size: *d* = 0.43; order of shouting [later]: *p* = 0.031; effect size: *d* = 0.48) (Fig. 4): shouting significantly increased the handgrip force level of sustained MVC.

**Table 2:** Repeated-measures three-way analyses of variance (ANOVAs) results for maximal voluntary force.

Source of variation	Degree of freedom	F-value	p-value	Partial *η*^2^
Group	1	0.022	0.88	0.002
Error	14			
Set	4	56.8	4.44 × 10^–19^	0.80
Set × group	4	1.22	0.30	0.081
Error	56			
Condition	1	0.056	0.81	0.004
Order × group	1	13.2	0.003	0.48
Error	14			
Set × order	4	14.0	5.58 × 10^–8^	0.50
Set × order × group	4	0.31	0.86	0.022
Error	56

### TMS

**Figure 5:**
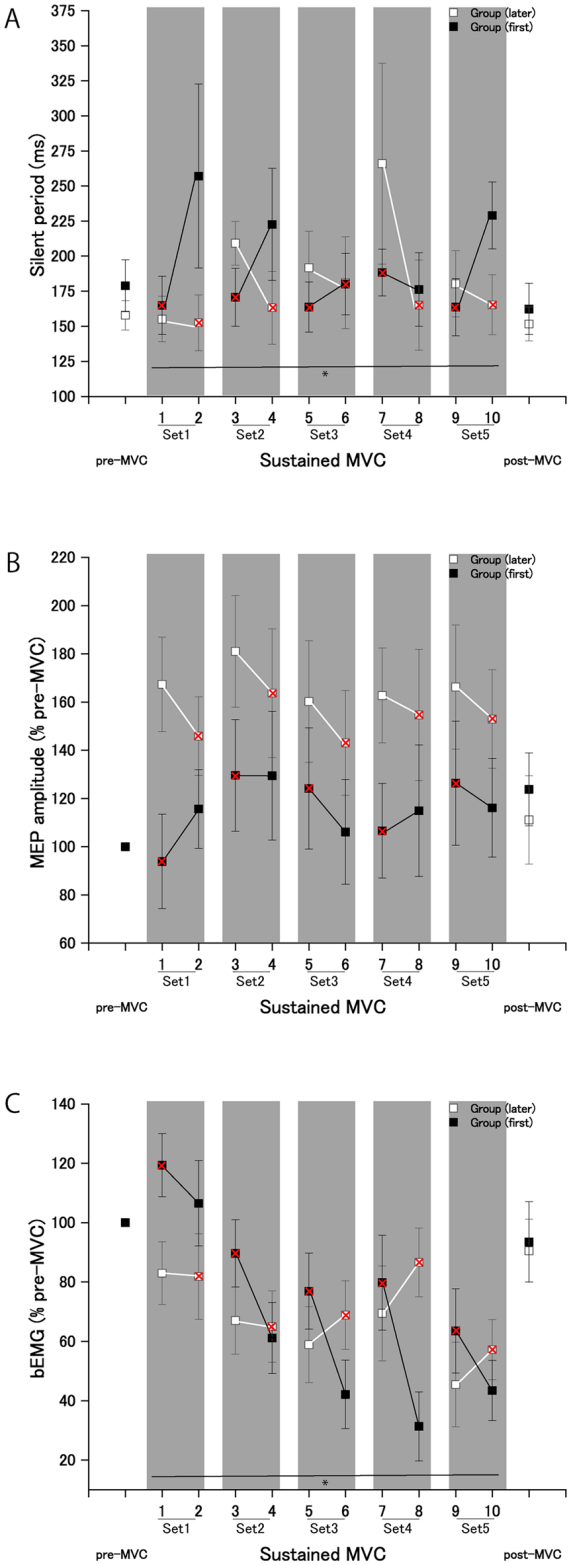
Effects of shouting on the silent period, motor evoked potential (MEP) amplitude and background (b) EMG. (**A**) Durations of the silent period for each condition (pre-sustained MVC, sustained MVC, and post-sustained MVC conditions) during the maximal voluntary contraction (MVC) of handgrip. (**B**) Amplitudes of MEPs of the flexor carpi ulnaris (FCU) for the three conditions (pre-sustained MVC, sustained MVC, and post-sustained MVC conditions) during the handgrip MVC. (**C**) Background (b) EMG activity of the FCU for the three conditions (pre-sustained MVC, sustained MVC, and post-sustained MVC conditions) during the handgrip MVC. Shouting significantly shortened the duration of the silent period, and significantly increased bEMG during a 2-min sustained MVC. A red cross indicates handgrip contraction with shouting. *Statistically significant difference between the contractions performed with and without shouting in the shaded area. Data are expressed as the mean ± standard error of the mean.

Compared with no shouting, the duration of the silent period during handgrip MVC was reduced when combined with shouting. Three-way ANOVA revealed that there was no significant second-order interaction (*F*[4,56] = 0.47; *p* = 0.75; effect size: η^2^_p_ = 0.03) (Table 3). There were significant first-order interactions between the order of each set (shouting or no shouting) and experimental group (*F*[1,14] = 17.09; *p* = 0.001; effect size: η^2^_p_ = 0.55). However, the MEP amplitude revealed no significant changes between the two orders (shouting vs. no shouting), as shown in Table 4. A three-way ANOVA revealed that there was no significant second-order interaction concerning the bEMG (*F*[2.32,32.6] = 1.61; *p* = 0.21; effect size: η^2^_p_ = 0.10) (Table 5). There were significant first-order interactions between the order of each set (shouting or no shouting) and experimental group (*F*[1,14] = 11.54; *p* = 0.004; effect size: η^2^_p_ = 0.45) and between the set (sets 1–5) and experimental group (*F*[4,56] = 3.14; *p* = 0.021; effect size: η^2^_p _= 0.18) (Fig. 5). Significant changes in the bEMG during sustained MVC were observed when combined with shouting in the same manner as observed for the maximal voluntary force. An analysis of covariance with bEMG as a covariate revealed that there was no significant interaction between the set (sets 1–5) and bEMG (F[9,140] = 1.25; *p* = 0.26; effect size: η^2^_p_ = 0.07); however, there was a significant difference in the y-intercept (p < 0.001).

**Table 3:** Repeated-measures three-way analyses of variance (ANOVAs) results for silent period.

Source of variation	Degree of freedom	F-value	p-value	Partial *η*^2^
Group	1	0.068	0.79	0.005
Error	14			
Set	2.15	0.43	0.66	0.003
Set × group	2.15	2.11	0.13	0.13
Error	30.1			
Order	1	0.16	0.69	0.012
Order × group	1	17.0	0.001	0.55
Error	14			
Set × order	4	3.21	0.019	0.18
Set × order × group	4	0.47	0.75	0.033
Error	56

**Table 4:** Repeated-measures three-way analyses of variance (ANOVAs) results for MEP amplitude.

Source of variation	Degree of freedom	F-value	p-value	Partial *η*^2^
Group	1	2.26	0.15	0.13
Error	14			
Set	4	1.60	0.18	0.10
Set × group	4	0.25	0.90	0.018
Error	56			
Order	1	3.06	0.102	0.17
Order × group	1	3.34	0.089	0.19
Error	14			
Set × order	4	0.80	0.52	0.054
Set × order × group	4	1.00	0.41	0.067
Error	56

**Table 5:** Repeated-measures three-way analyses of variance (ANOVAs) results for bEMG.

Source of variation	Degree of freedom	F-value	p-value	Partial *η*^2^
Group	1	0.066	0.80	0.005
Error	14			
Set	4	9.67	5 × 10^–5^	0.40
Set × group	4	3.14	0.021	0.18
Error	56			
Order	1	4.23	0.059	0.23
Order × group	1	11.5	0.004	0.45
Error	14			
Set × order	2.32	0.40	0.69	0.028
Set × order × group	2.32	1.61	0.21	0.10
Error	32.60

### Pupil area

A three-way ANOVA revealed that there was no significant second-order interaction or significant first-order interaction (Table 6). However, there was a significant main effect of the order of each set (shouting vs. no shouting) on the pupil area (*F*[1,14] = 8.86; *p* = 0.01; effect size: η^2^_p_ = 0.38). Post hoc analyses with Bonferroni corrections for multiple comparisons demonstrated that there was a significant change between two orders (shouting vs. no shouting) at time point 3 (*t*[16] = 3.25; effect size: *d* = 0.63; *p* = 0.005).

**Table 6:** Repeated-measures three-way analyses of variance (ANOVAs) results for pupil area.

Source of variation	Degree of freedom	F-value	p-value	Partial *η*^2^
Group	1	2.99	0.10	0.17
Error	14			
Set	2.50	1.60	0.21	0.10
Set × group	2.50	0.41	0.70	0.029
Error	35.0			
Order	1	8.86	0.01	0.38
Order × group	1	0.24	0.62	0.17
Error	14			
Set × order	2.26	0.84	0.45	0.057
Set × order × group	2.26	0.90	0.42	0.061
Error	31.7			

## Discussion

The results reveal that shouting significantly increased the handgrip force level of sustained MVC, as a result of a reduction in the duration of the silent period. Such an enhancing effect of shouting on handgrip MVC is generally consistent with the results of previous studies^1,2,5^. Our findings indicate that the motor system was more excitable during sustained muscular contraction paired with shouting, leading to the generation of additional muscular force in maximal exertion effort. These results show that the muscular force-potentiating effect of shouting in maximal force exertion is relevant to the potentiation of motor system activity, via the additional drive of shouting operating on M1.

The main finding was the remarkable potentiation effect of shouting on the handgrip maximal voluntary force during a 2-min period of sustained MVC, with an average increase of approximately 30% in the handgrip force. This increase is greater than the effect of shouting on the maximal voluntary force (15%) during a brief MVC reported in our previous study^5^. One potential reason for this discrepancy is a difference in the experimental period of muscular force exertion between the present study (2 min) and the previous study (within 5 s). It is conceivable that maximal force exertion for 2 min induces both peripheral and central fatigue, resulting in a marked decline in force production during a 2-min sustained MVC. Indeed, the handgrip maximal voluntary force declined to approximately 70% of that for pre-sustained MVC. This could be due to a progressive, exercise-induced decline in the voluntary activation of a muscle during sustained maximal effort, which relates to impairment at sites proximal to the neuromuscular junction^6^. Thus, the differences in the potentiating effects on MVC mentioned above may be relevant to the experimental period and fatigue of human voluntary contractions.

The muscular force-enhancing effect of shouting was accompanied by a reduced duration of the silent period. Changes in the durations of silent periods of longer than 100 ms in the hand muscles of healthy participants^20^ are considered to reflect cortical inhibition^15^. The cortical silent period originates largely from M1^15^, where GABAergic circuits are believed to produce the cortical silent period^21–23^. The cortical silent period is used as a measure for assessing motor excitability^24^. Thus, shouting transiently may enhance the activity of motor cortical neurons. At the same time, we must keep in mind that shouting may enhance the activity of spinal motor neurons, because the bEMG during handgrip MVC significantly increased when combined with shouting. However, we were convinced that such an enhancement of the spinal motor neuronal activity may be caused by the enhancing effect of shouting on motor cortical neurons because an analysis of covariance with bEMG as a covariate revealed that there was no significant interaction between the set (sets 1–5) and bEMG (F [9,140] = 1.25; *p* = 0.26; effect size: η^2^_p _= 0.07); however, there was a significant difference in the y-intercept (p < 0.001). Thus, there was a significant difference in the duration of the silent period between with and without shouting, irrespective of changes in the bEMG. This notion is supported by recent evidence that shouting increases handgrip MVC through the reduction of motor cortical inhibition with no significant changes in the bEMG^5^.

Such a statistically significant increase in the bEMG during handgrip MVC when combined with shouting might be due to the shouting-induced increase in the voluntary drive to the motor cortex (Fig. 5C). Meanwhile, there were no statistically significant differences in the bEMG during handgrip contraction between with (mean ± standard error: 12.20 ± 2.79 μV s) and without shouting (11.59 ± 2.65 μV s) in a previous study^5^ (see Fig. 2B in the previous study; *t*[18] = − 1.03, effect size: *d* = 0.24; *p* = 0.31). The difference in the bEMG between the current study and the previous study^5^ may relate to whether the force exertion was followed by shouting; i.e., the sustained handgrip MVC during the period approximately 100 ms before TMS was accompanied by shouting because the participants in the present experiment were asked to shout as loudly as possible for 1–2 s in a 2-min sustained MVC with five periods of intermittent shouting. This speculation is supported by the results of a previous study^25^, where fatiguing muscular contraction increased the duration of the silent period with unchanged motoneuronal responsiveness.

We found that shouting led to a reduced duration of the silent period and increased handgrip maximal voluntary force. The increased maximal voluntary force may have resulted from the reduced motor cortical inhibition. These enhancement effects of shouting on handgrip MVC and motor system activity were observed when participants shouted while exerting the handgrip muscular force during maximal effort; i.e., there was no shouting immediately prior to or almost simultaneously with force exertion. Thus, the additional drive of shouting operating on the motor system led to a reduced duration of the silent period and increased maximal voluntary force. This result supports the present finding that producing a shout without handgrip muscular contraction enhanced the motor system state, indicating that the motor command for shouting is transmitted to the motor cortex, and in the final cortical stage of the motor execution program the motor command of shouting is devoted to M1. Therefore, shouting-induced excitatory input to M1 contributes to the muscular force-enhancing effect of shouting during maximal force exertion.

Despite the reduced duration of the silent period, there were no statistically significant differences in MEP amplitudes between with and without shouting during handgrip MVC (Fig. 5B). The reason for this failure to detect any changes is that most of the M1 neurons have already been recruited^26^, leaving fewer neurons available to respond to TMS. Thus, the level of M1 neuron recruitment reaching a plateau during MVC might have overshadowed any differences in MEP amplitudes during MVC between with and without shouting.

**Figure 6:**
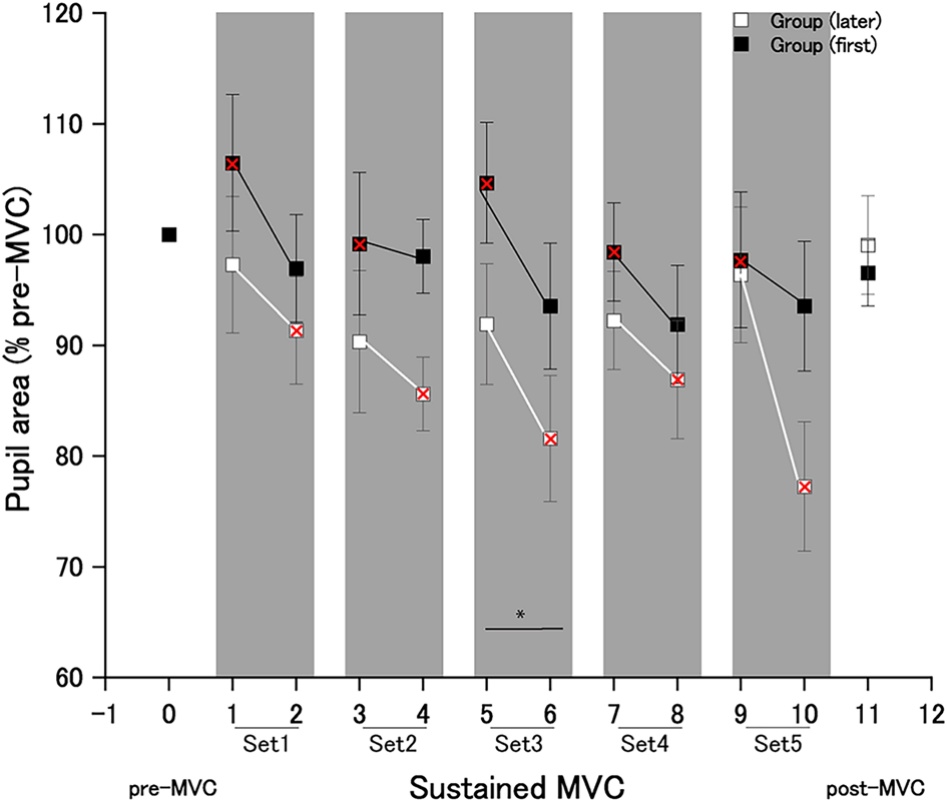
Effects of shouting on pupil area. Averaged pupil area (dots) for 500 ms before each transcranial magnetic stimulation during squeezing a handgrip device in the pre-sustained MVC, post-sustained MVC, and sustained MVC conditions in each experimental group. Red cross indicates handgrip contraction with shouting. *Statistically significant difference between the handgrip contractions performed with and without shouting in shaded area. Data are expressed as the mean ± standard error of the mean.

A recent study reported that shouting significantly increased the handgrip force level of a brief MVC for 1–2 s and was thus followed by an increase in the pupil size^5^. However, pupillary dilation can result from causes other than self-generated shouting; e.g., motor imagery when performing the handgrip MVC combined with a self-generated shout^5^ (see the “Discussion” section of the previous study). Pupillary dilation was not observed during the handgrip MVC phase but was observed during the experimental instruction phase, in which participants viewed the experimental instructions on force exertion and shouting in the resting state (without muscular contraction) immediately before the handgrip MVC phase with shouting. Therefore, we cannot exclude the possibility that pupil-dilating effects during the experimental instruction phase affected pupillary dilation during the handgrip MVC phase with shouting. To ensure this did not occur in the current study, a pre-sustained MVC condition equivalent to the experimental instruction phase of the previous study^5^—handgrip muscular contraction without shouting—was adopted so as not to observe the handgrip MVC combined with a self-generated shout, at least 3 min apart from the 2-min-sustained MVC condition. The difference of more than 30% in the mean pupil area between the experimental instruction phase (control condition: 1663.8 ± 65.7 dots; shout condition: 1822.0 ± 98.2 dots) in the previous study^5^ (see Fig. 5A,B in the previous study) and the pre-sustained MVC condition (Experimental group [later]: 2807.0 ± 159.2 dots; Experimental group [first]: 2401.0 ± 171.4 dots) in the current study may be mainly due to whether there was handgrip MVC. We expected shouting-induced pupillary dilation under the 2-min-sustained MVC condition via alteration of the state of pupil-linked neuromodulatory systems through enhancement of pathways necessary for the production of shouting^5^. However, no consistent enhancing effects of shouting during handgrip MVC on the pupil area were observed in the present study (see “Pupil area” in “Results” for details). One reason for this discrepancy is a difference in the experimental protocol concerning MVC between the present study (continuous) and previous study (momentary). It is conceivable that there is no room for pupillary dilation when shouting is performed throughout maximal force exertion for 2 min. In the current study, the pupil area markedly increased immediately after the appearance of the word “squeeze” on the display and remained at a high level until the end of squeezing; there was no significant difference in pupil area between pre-sustained MVC and sustained MVC without shouting (Fig. 6; Table 1: Set 1 no-shout in order of shouting [first]) (*t*[7] = 0.96, *d* = 0.34; *p* = 0.36). Thus, the differences in the effect of shouting on the pupil area may be related to experimental force exertion protocol in maximal effort. It is noted, however, that the current results do not necessarily imply that shouting never affects the pupil-linked neuromodulatory system state. The level of pupillary dilation reaching a plateau during MVC^27^ might have overshadowed any differences in the pupil-linked neuromodulatory system state between with and without shouting.

We found that shouting led to a reduced duration of the silent period during MVC and increased handgrip maximal voluntary force. The increased handgrip maximal force may be due to the reduced motor cortical inhibition during maximal effort, through the additional drive of shouting operating on the motor system. These results indicate that the muscular force-potentiating effect of shouting in maximal force exertion is relevant to the potentiation of motor system activity, caused by the transmitted motor command of shouting to M1. In turn, this suggests that maximum volition does not cause the motor system to generate maximum activity. This is an active characteristic of the neural systems in the human brain. However, we do not know to what exact extent that spinal motor neuronal excitability is involved in neural activity of the force-enhancing effect of shouting, a puzzle that remains to be solved. From the perspective of accessing the latent ability for human force exertion, it may be useful for future studies to examine whether devoting an additional motor command, such as shouting, to M1 improves the characteristics of neural system activity. One limitation of the current study is small sample size (eight per experimental group [later or first]), following that there were the three samples out of one hundred samples (two experimental groups × ten trials [MEPs] × five measurements) in Figs. 4, 5, and 6, of which the data did not follow a normal distribution using the Kolmogorov–Smirnov test: the duration of the silent period in Experimental group(later) set 4 no-shout, MVC in Experimental group(later) set1 no-shout and Experimental group(first) set 3 no-shout. Thus, a potentially important limitation of our study is the small number of participants in each experimental group, providing careful interpretation of interaction effects. However, it is reasonable to assume that a self-generated shout following force exertion in the sustained fatiguing maximal effort enhances the maximal voluntary force through potentiation of the motor system. This is because our results in the present study basically agree with those in the previous study^5^ that shouting increased the handgrip maximal voluntary force during brief non-fatiguing maximal effort through potentiation of motor system, based on the same idea that producing a shout in itself is a requisite for the muscular force-enhancing effect of shouting. At the same time, we must keep in mind that future studies are needed to confirm our findings by the study with the large number of participants in each group.

## Data Availability

All data generated or analyzed during this study are included in this published article.
